# Cancer-testis antigens in ovarian cancer: implication for biomarkers and therapeutic targets

**DOI:** 10.1186/s13048-018-0475-z

**Published:** 2019-01-04

**Authors:** Kaipeng Xie, Chenyang Fu, Suli Wang, Hanzi Xu, Siyu Liu, Yang Shao, Zhen Gong, Xiaoli Wu, Bo Xu, Jing Han, Juan Xu, Pengfei Xu, Xuemei Jia, Jiangping Wu

**Affiliations:** 1 The Affiliated Obstetrics and Gynecology Hospital of Nanjing Medical University, Nanjing Maternity and Child Health Care Hospital, Women’s Hospital of Nanjing Medical University, Nanjing, 210004 China; 2 Jiangsu Institute of Cancer Research The Affiliated Cancer Hospital of Nanjing Medical University, Nanjing, 210009 China; 3 State Key Laboratory of Reproductive Medicine, Institute of Toxicology, Nanjing Medical University, Nanjing, 211166 China

**Keywords:** Cancer-testis antigens, Ovarian cancer, Tumor development, Immunotherapy

## Abstract

Ovarian cancer remains the most fatal gynecologic malignancy worldwide due to delayed diagnosis as well as recurrence and drug resistance. Thus, the development of new tumor-related molecules with high sensitivity and specificity to replace or supplement existing tools is urgently needed. Cancer-testis antigens (CTAs) are exclusively expressed in normal testis tissues but abundantly found in several types of cancers, including ovarian cancer. Numerous novel CTAs have been identified by high-throughput sequencing techniques, and some aberrantly expressed CTAs are associated with ovarian cancer initiation, clinical outcomes and chemotherapy resistance. More importantly, CTAs are immunogenic and may be novel targets for antigen-specific immunotherapy in ovarian cancer. In this review, we attempt to characterize the expression of candidate CTAs in ovarian cancer and their clinical significance as biomarkers, activation mechanisms, function in malignant phenotypes and applications in immunotherapy.

## Introduction

Ovarian cancer is one of the most fatal gynecologic malignancies worldwide, with an estimated incidence of 239,000 cases and 152,000 deaths each year [1]. According to the latest data from the National Central Cancer Registry of China and The American Cancer Statistics, the ratios of new cases and deaths were 2.93% and 2.24%, respectively, in China and 2.6% and 5.06%, respectively, in America [2]. During the past decade, an increasing trend in mortality has been observed for ovarian carcinoma in China [3]. Due to delayed diagnosis, tumor recurrence and chemotherapy resistance, the 5-year survival rate of patients with advanced disease was approximately 30% [2]. Hence, there is an urgent need to explore new avenues for early diagnosis, prognosis and therapeutic targets for ovarian cancer.

With the development of genome-wide sequencing in the last decade, the genomic landscape of ovarian cancer has been gradually unveiled [4, 5]. Large-scale genomic projects, such as The Cancer Genome Atlas (TCGA), have identified driver mutations in ovarian carcinogenesis and have provided new strategies for specific targeted therapies [4–6]. However, only a few mutations in these mutation-driver (mut-driver) genes are able to induce tumorigenesis [7, 8]; most of these mutations are present at low population frequencies and in a small proportion of patients [4, 8]. Notably, Vogelstein *et al* proposed that driver genes include not only mut-driver genes but also epi-driver genes, which are aberrantly expressed in tumors but not frequently mutated; they are altered through changes in DNA methylation or chromatin modification that persist as the tumor cell divides [8]. Among these genes, cancer-testis antigens (CTAs) or cancer-testis (CT) genes have attracted particular attention due to their restricted expression patterns and immunogenicity [9, 10].

CTAs consist of a cluster of genes exclusively expressed in normal testis tissues, yet abundant levels are found in several types of tumor tissues. The role of CT genes in spermatogenesis and tumorigenesis is an interesting issue. Reactivation of normally silent CT gene expression in cancer cells may confer some of the central characteristics of tumor formation and progression, including demethylation, angiogenesis induction, major histocompatibility complex downregulation (immune evasion), and chorionic gonadotropin expression [10]. The similarity between gametogenesis and tumorigenesis includes the following: immortalization of primordial germ cells and transformation of tumor cells; ploidy cycles in meiosis and aneuploidy in tumor cells; migration of primordial germ cells and metastasis of tumor cells. Emerging studies have suggested that aberrant expression of CTAs may drive soma-to-germline transformation and result in tumorigenesis and tumor progression [11, 12]. Owing to their immunogenic properties, these germ-line genes may represent novel targets for cancer immunotherapy [13]. Indeed, several CTAs have become a major focus for the development of vaccine-based clinical trials in recent years. For example, combined analysis of NY-ESO-1-based trials revealed that patients with NY-ESO-1-positive tumors who received immunotherapy exhibited a 2-year overall survival (OS) advantage compared with those with NY-ESO-1-positive tumors who did not receive vaccination [14]. Given the increasing number of identified CT genes and the recent research advances in ovarian cancer, we herein summarize the characteristics, clinical applications, potential activation mechanisms and function of CTAs in ovarian cancer.

## Expression of CT genes in ovarian cancer

CTAs are classified into two categories: CT-X antigens located on the X chromosome and non-X CTAs located on autosomes [15]. The first cancer antigen MAGE-A1 was recognized by autologous cytotoxic T lymphocytes in a melanoma patient [16, 17]. Subsequent advances in biotechnology have enabled scientists to explore more cancer antigen subgroups expressed in gametogenic tissues and a variety of tumors, including multiple myeloma [18] and ovarian cancer [19]. The list of identified CT genes has expanded to approximately 250 genes in the CT database (http://www.cta.lncc.br) [20]. Based on multiple independent large databases, including Genotype-Tissue Expression (GTEx), and human proteomic and TCGA data, our group recently systematically explored the molecular landscape of CT genes in 19 cancer types [9]. CTA family members in ovarian cancer reported to date include MAGE genes, NY-ESO-1, SSX, and CT45, which are categorized as CT-X antigens, and BORIS, PRAME, PIWIL, and AKAP3/4, which are categorized as non-X CTAs. A growing number of studies have focused on the identification of CTAs in ovarian cancer. The characteristics of CTAs are summarized in Table 1.

**Table 1:** Characteristics of cancer-testis antigens (CTAs) in ovarian cancer (OC)

CT genes	Location	Expression in OC	Positive ratio in tissues	Clinical significances in OC	Molecular biological functions involved in OC	References
AKAP3	12p13.32	OC tissues	mRNA 58% (43/74), 28% (15/54)	OS, PFS, Grade, Stage	Cell migration, Invasion	[28, 42, 69]
		SVOV3, SKOV3, OV432				
AKAP4	Xp11.22	OC tissues	mRNA & protein 89% (34/38)	NR	ROS generation, DNA damage, Cell cycle, Apoptosis, EMT	[29, 70]
		A10, Caov-3, SKOV3				
BAGE	21p11.2	OC tissues	mRNA 14.6% (6/41)	Metastasis, Diagnostic sensitivity in ascites	NR	[24, 37]
		OC ascites specimens	**mRNA 63% (17/27)**			
		COC1				
BORIS	20q13.31	OC tissues	NR	OS, PFS, Stage, global DNA hypomethylation	Immortalization (Activates hTERT transcription) Coregulation with other CT genes	[49, 51, 58, 94]
		OVCAR3, A2780, OVCAR429				
CT45	Xq26.3	OC tissues	mRNA 25% (29/118)	OS, DFS, Stage	Coregulation with other CT genes	[27, 31]
		OVSAHO	Protein 37% (82/219)			
GAGE-1/2	Xp11.23	OC tissues	mRNA 26.8% (11/41)	Diagnostic sensitivity in ascites	Paclitaxel-resistant phenotype	[24, 34, 37, 78]
		OC ascites specimens	**mRNA 30% (8/27)**			
		A2780	Protein 13.9% (30/216)			
HOM-TES-85	Xq23	OC tissues	mRNA 32% (7/22)	NR	Transcriptional activity and splicing	[95]
HORMAD1	1q21.3	Cell lines^a^	NR	NR	Cell survival, Invasion, Migration, Tumor microenvironment (VEGF, NF-κB)	[76, 77]
HSP70-2	6p21.33	SKOV3, Caov-3, A-10	NR	NR	Cell cycle, Apoptosis, EMT	[67]
LAGE-1	Xq28	OC tissues	mRNA 21% (22/107)	Stage	NR	[25]
		SKOV3				
MAGE-A1	Xq28	OC tissues	mRNA 53.7% (22/41), 21% (12/58), 40.3% (25/62)	PFS, Tumor differentiation, Stage, Metastasis, Diagnostic sensitivity in ascites	Cell proliferation, Migration (NOTCH signaling), Coregulation with other MAGE genes.	[19, 21, 24, 37, 78, 96]
			mRNA or protein 15% (42/281)			
		OC ascites specimens	**mRNA 7% (2/27)**			
		Cell lines^b^				
MAGE-A2	Xq28	OC tissues	mRNA 9% (5/58)	Chemotherapy	Cell growth, Paclitaxel-resistant phenotype, Coregulation with other MAGE genes.	[19, 78]
MAGE-A3	Xq28	OC tissues	mRNA 36.6% (15/41), 40.3% (25/62)	Tumor differentiation, Stage,	Cell growth, Coregulation with other MAGE genes,	[19, 21, 23, 33, 37, 78]
			mRNA or protein 36% (131/390)	Diagnostic sensitivity in ascites or plasma	Paclitaxel-resistant phenotype	
		OC ascites specimens	**mRNA 30% (8/27)**			
		A2780, COC1, OV-90				
MAGE-A4	Xq28	OC tissues	mRNA 7% (4/58), 64.5% (40/62)	OS	Coregulation with other MAGE genes	[19, 21, 22, 33, 34]
			Protein 23% (49/216)			
			mRNA or protein 47% (186/399)			
		Serum	**Protein 22% (13/60)**			
MAGE-A9	Xq28	OC tissues	Protein 37% (47/128)	OS, Stage, Grade, CA-125, Metastasis	NR	[39]
MAGE-A10	Xq28	OC tissues	mRNA 11.3% (7/62)	PFS	Coregulation with other MAGE genes	[21, 33]
			mRNA or protein 52% (204/395)			
MAGE-C1	Xq27.2	OC tissues	mRNA 14.5% (9/62)	PFS, Grade, Chemotherapy	Coregulation with other MAGE genes	[21, 33, 34]
			mRNA or protein 16% (42/267)			
			Protein 25% (52/212)			
NY-ESO-1	Xq28	OC tissues	mRNA 25.9% (260/1002)	OS, PFS, Stage, Grade	Coregulation with other CT genes ( LAGE-1 )	[14, 25, 33, 34]
			mRNA 30% ( 32/107) 8.1% (5/62)			
			Protein 43% (62/143), 7.1 % (15/211), 26.5% (232/874)			
		SKOV3, A2780, OVCAR3	mRNA or protein 41% (408/1002), 43% (82/190)			
OY-TES-1	12p13.31	OC tissues	mRNA 32.3% (20/62)	Survival time, faster relapse	Spindle-pole congression, Cell proliferation, Paclitaxel resistance	[33, 35, 83, 97]
			mRNA or protein 69% (69/100)			
		PEO4, PEO1	Protein 81% (87/107)			
PRAME	22q11.22	OC tissues	mRNA 60% (70/119)	OS, DFS, Grade, Stage, Metastasis,	NR	[60, 98]
		Cell lines^c^		Global DNA hypomethylation		
PASD1	Xq28	OC tissues	NR	NR	NR	[99]
PIWIL1	12q24.33	OC tissues	mRNA 61% (129/211)	OS, Metastasis, Chemotherapy,	Interacts with let-7a, Invasiveness, Angiogenesis	[43, 100–102]
		A2780	Protein 18/20 (90%)			
PIWIL2	8p21.3	OC tissues	mRNA 29% (18/62)	Metastasis	Cisplatin resistance (repair of cisplatin-induced DNA damage)	[33, 43, 82]
		Cell lines^d^	Protein 19/20 (95%)			
PIWIL3	22q11.23	OC tissues	Protein 19/20 (95%)	Metastasis		[43]
PIWIL4	11q21	OC tissues	Protein 19/20 (95%)	Metastasis		[43, 102]
		OVCAR5				
PLU-1	1q32.1	OC tissues	Protein 71% (85/120)	OS, DFS, Stage, Grade, Metastasis, Chemotherapy	NR	[80]
PLAC1	Xq26.3	OC tissues,	mRNA 21% (21/101)	NR	NR	[61, 103, 104]
		Cell lines ^e^				
SPAG9	17q21.33	OC tissues	mRNA 90% (18/20)	NR	Cell proliferation, Invasion, Migration (JLP-JNK signaling)	[30]
		Cell lines^f^	Protein 67% (20/30)			
SCP-1	1p13.2	OC tissues	mRNA 15% (15/100)	Grade, survival	NR	[105]
SP17	11q24.2	OC tissues	mRNA 68% (17/25)	Chemotherapy	Cell migration, Chemosensitivity (carboplatin, cisplatin).	[32, 91, 106]
			Protein 43% (30/70)		Vaccination against SP17 controlled tumor growth in a murine model	
		Metastatic ascitic fluid				
		HO-8910, ES-2, SKOV-3				
SSX2	Xp11.22	OC tissues,	mRNA 10% (12/122)	Potential diagnostic markers for ovarian cancer (anti-SSX common epitope, sera)	NR	[26, 107]
		SVOV3, OVCAR3,				
SSX1	Xp11.22	OC tissues	mRNA 2.5% (/3/118)			
		SKOV3				
SSX4	Xp11.22	OC tissues,	mRNA 16% (19/120)			
		SVOV3, SKOV3, OVCAR3	mRNA 50% (6/12)			
TAG-1	5p15.2	OC tissues	mRNA 35% (8/23)	NR	NR	[108]
		Cell lines^g^				
TAG-2a	5p15.2	OC tissues	mRNA 22% (5/23)			
		Cell lines^h^				
TAG-2b	5p15.2	OC tissues	mRNA 9% (2/23)			
		OVCAR3, TTB-6				
TAG-2c	5p15.2	OC tissues	mRNA 9% (2/23)			
		OVCAR3, SW626, TTB-6				
TRAG-3	Xq28	OC tissues	Protein 83.8% (31/37)	OS, PFS, Chemotherapy	Coregulation with other cancer-testis antigens.	[36, 81]
		SKOV-3TR, OVCAR-4				

OC, Ovarian cancer; OS, Overall survival; PFS, Progression-free survival; EMT, Epithelial-mesenchymal transition; ROS, Reactive oxygen species; DFS, Disease-free survival

NR, Not reported. ^a^A2780, 2774, SKOV3, ES-2. ^b^ SKOV3, A2780, COC1, SKOV3ip. ^c^OVCAR3, SKOV3, CAOV-3, OVSAHO, A2780, SNU119, IOSE121

^d^OVCAR5, CDDP, 2008C13, A2780, CP70, MCP2, MCP3, MCP8, 2008. ^e^ CAOV4, SKOV3, CaoV3, BG1

^f^A-10, SKOV-6, CAOV-2, Cov362, OVCAR4, Cov318, CAOV-3, OV90, ES-2, TYKNU

^g^CAOV-3, CAOV-4, ES-2, OV-90, OVCAR3, SKOV3, SW626, SW626, TOV-21G, TTB-6. ^h^CAOV-3, COV413, OV-90, OVCAR3, SW626, TTB-6

Bold font indicates the positivity rate in bodily fluids rather than in tissues

As one classical type of CT gene, the expression levels of MAGE genes in ovarian cancer have been widely investigated, e.g., the frequency of MAGE-A1 mRNA expression was found to be 20.7% among 58 ovarian cancer tissues [19]. Moreover, Daudi *et al* examined MAGE expression in 400 epithelial ovarian cancer (EOC) tissues by reverse transcription-polymerase chain reaction (RT-PCR) and immunohistochemistry (IHC) and found that at least one of five MAGE antigens (MAGE-A1, MAGE-A3, MAGE-A4, MAGE-A10 and MAGE-C1) was expressed in approximately 78% of EOC patients [21]. Another study further demonstrated that MAGE-A4 serum levels were significantly increased in ovarian cancer patients compared with those with benign diseases, and the MAGE-A4 protein was expressed in 22% of primary ovarian cancer patients [22]. One interesting finding is that MAGE-A3/6 protein was found to be present on plasma-derived exosomes from all ovarian cancer patients assessed but not on those from benign tumors or healthy controls [23]. Coincidentally, Hofmann and colleagues detected BAGE, MAGE-A1, MAGE-A3 and GAGE1/-2 mRNAs in peritoneal fluid from ovarian cancer patients using multiplex RT-PCR analysis, with the combination of the four markers exhibiting increased diagnostic sensitivity of 94% compared with cytomorphology alone [24].

Odunsi *et al* reported that either NY-ESO-1 or LAGE-1 was expressed in approximately 50% of 107 EOC specimens [25], and subsequent CTA analysis in a large cohort of ovarian cancer patients (n=1002) revealed NY-ESO-1 expression by any method in 41% of tumors [14]. In addition, aberrant expression of SSX-1, SSX-2, or SSX-4 was identified in 26% of ovarian tumor specimens, and SSX-1, SSX-2, and SSX-4 expression was detected in 2.5%, 10%, and 16% of 120 EOC specimens, respectively [26]. Among 219 ovarian cancer cases included in a tissue microarray, CT45 was expressed in 82 samples (37%), with the majority of cases showing moderate (++) to strong (+++) expression [27]. Furthermore, high AKAP3 mRNA expression was observed in 43/74 (58%) of ovarian cancer specimens [28]. Although AKAP4 mRNA and protein were not expressed in 21 matched adjacent non-cancerous tissues, they were detected in 89% (34/38) of ovarian carcinoma tissues [29]. One study reported extensive distribution of SPAG9 mRNA and protein in 90% (18/20) of EOC tissue specimens [30]. CT45 is reportedly not expressed in normal ovary samples but is activated in a significant proportion of EOC specimens (25%), with marked expression in serous EOC but not in non-serous EOC [31]. It has also been reported that sperm protein antigen 17 (Sp17) is expressed in adenocarcinoma cells of all epithelial ovarian cancer subtypes and in 43% (30/70) of EOC cases [32]. Recently, findings from Garcia-Soto *et al* suggest that CT genes (a panel of 20 CT genes) are expressed in 95% of EOC tumor specimens, with a mean number of 4.5 [33].

Notably, emerging evidence indicates that CT genes are preferentially expressed in a cluster, indicating that these genes may share common regulatory elements and possible functional relevance. One study suggested that NY-ESO-1 and LAGE-1 are dually expressed in 11% of epithelial ovarian tumor specimens as well as the SKOV3 cell line [25]. MAGE-A4 exhibits a relatively high expression frequency and plays a central role in the co-expression of other MAGE antigens [21]. MAGE-C1 expression and co-expression of CT genes are significantly correlated with the grade of endometrioid ovarian cancer and the histological subtype [34], and analysis of Oncomine datasets has confirmed that CT45 is co-expressed with other CT genes in EOC, including MAGE and GAGE genes [31]. Importantly, a subset of CT genes shows distinct expression in specific pathological subtypes or different stages of ovarian cancer; some CT genes are likely expressed in serous histologic subtypes. The majority of MAGE-mRNA-positive ovarian tumors are histologically classified as surface-epithelial-stromal tumors, particularly serous adenocarcinomas [19]. One study found that AKAP4 was expressed in serous adenocarcinoma and serous papillary carcinoma but not in endometrioid adenocarcinoma or adenofibroma [29]. Patients with late-stage or high-grade EOC often express increased CT45 levels [31]. Expression of TRAG3 is also increased after the initiation of chemotherapy following initial surgery [36]. Taken together, because of their unique characteristics, CT genes are promising biomarkers for diagnosing ovarian cancer and assessing tumor aggressiveness.

## Tumor progression and prognosis

An increasing number of studies have reported correlations between CT genes and tumor progression and prognosis. Regarding MAGE members, expression of MAGE-A1 and MAGE-A3 correlates with tumor differentiation and clinical stage in ovarian cancer [37]; MAGE-A1 and MAGE-A10 expression is also significantly associated with poor progression-free survival (PFS) in EOC [21]. MAGE-A4 is a reliable prognostic factor for serous carcinoma patients [38]. MAGE-A9 expression was also found to be significantly associated with high histological grade, International Federation of Gynecology and Obstetrics (FIGO) stage, CA-125 level and metastasis. In addition, patients with MAGE-A9 expression exhibited poor OS [39]. Consistently, one study examined the prognostic significance of MAGE-A family expression in EOC patients and showed MAGE-A expression to be associated with pathological type, FIGO stage, and pre-operative serum CA125 level. Furthermore, the OS of EOC patients with MAGE-A family expression was significantly reduced compared with patients negative for MAGE-A expression [40]. In addition, the expression levels of MAGE-As and each individual MAGE-A gene in peripheral blood were associated with low OS in ovarian cancer patients [41]. Enzyme-linked immunosorbent assay (ELISA) results for serum MAGE antigen-specific antibodies revealed that the presence of a humoral immune response to any MAGE antigen predicted poor OS [21].

Recently, Szender *et al* first demonstrated the association between NY-ESO-1 expression and an aggressive ovarian cancer phenotype, reporting that NY-ESO-1^+^ patients had more serous histotypes and grade 3 tumors, exhibited a higher stage and were less likely to have a complete response to initial therapy. A trend toward reduced PFS and significantly reduced OS has been noted among NY-ESO-1^+^ patients [14]. Similarly, patients expressing AKAP-3 mRNA exhibit a significantly poorer OS than patients lacking AKAP-3 expression [28]. Consistently, AKAP-3 is primarily expressed in advanced-stage and poorly differentiated cancers [42]. Furthermore, PIWI proteins are upregulated in EOC tissues and associated with tumor metastasis [43]. Zhang *et al* found CT45 to be activated only in late-stage and high-grade disease, and increased CT45 expression was correlated with reduced OS and DFS in EOC [31]. Taken together, these findings indicate that CT genes have the potential to serve as prognostic markers for ovarian cancer patients.

## Molecular mechanisms of CT gene expression

Based on the tendency of co-expression noted in cancers, CT genes are thought to share a common mechanism of regulation at the transcriptional level [44]. A body of work indicates that epigenetic regulation is a key mechanism in the transcriptional regulation of CT genes [45]. According to the literature, the mechanism of CTA activation includes the following several categories.

## DNA hypomethylation in CT gene activation

It is reported that DNA methylation plays a dominant role in the epigenetic hierarchy governing CT gene expression. DNA hypomethylation coordinately affects CT gene promoters in EOC and correlates with advanced-stage disease. One study provided initial evidence that activation of MAGE-A1 in cancer cells is caused by promoter DNA hypomethylation [46]. After exposure to the DNA demethylating agent 5-aza-2'-deoxycytidine (DAC), human ovarian cancer cell lines display an pattern of upregulated for many CT genes [47, 48]. CT genes are coordinately expressed in EOC, and this expression pattern is associated with promoter DNA hypomethylation [49]. Additionally, Yao *et al* utilized sodium bisulfite sequencing of genomic DNA to provide direct evidence that methylation of TRAG-3 exon 2 and the promoter suppresses its transcription [50]. Intriguingly, several studies have suggested that expression levels of CT genes correlate with global genomic DNA hypomethylation in cancers [51, 52]. For example, Woloszynska-Read and co-workers reported that in ovarian cancer, DNA hypomethylation drives expression of BORIS, which is partly determined by the global DNA methylation status [51]. Similar interconnections have been observed for NY-ESO-1, MAGE genes and some other CT genes. Woloszynska-Read *et al* reported that both intertumor and intratumor NY-ESO-1 expression heterogeneity is associated with promoter methylation and global DNA methylation in EOC [52]. MAGEA11 expression is also associated with promoter and global DNA hypomethylation and activation of other CT genes [53]. And a link between promoter DNA methylation alterations and CT45 expression was validated in EOC in clinical samples and EOC cell models [31]. CT45 hypomethylation shows a strong correlation with LINE-1 hypomethylation, which is a good indicator of the level of global DNA methylation [31]. All evidence to date suggests that expression of these genes in tumors is dependent on aberrant DNA hypomethylation. Moreover, Ahluwalia *et al* identified DNA methyltransferase genes as a key component of the DNA methylation process; these genes are upregulated in cancers and maintain the state of methylation [54]. In addition, DNA methylation regulates nucleosome occupancy and participates in CT gene activation. Indeed, DNA methylation regulates nucleosome occupancy at the -1 position of MAGEA11, which was correlated with methylation of a Ets site and involved in the repression of MAGEA11 activity [53]. Thus, it appears that DNA methylation may cooperate with sequence-specific transcription factors to regulate gene expression.

## Histone modification and modulation of CT gene expression

Demethylation and re-expression of CT genes in tumors is generally correlated with losses in repressive histone marks and gains in activating histone marks [55, 56]. Treatment of A2780/cp70 with DAC and a histone deacetylase (HDAC) inhibitor resulted in a marked increase in expression of epigenetically silenced MAGE-A1 both *in vitro* and *in vivo* [57]. Woloszynska-Read *et al* observed that genetic disruption of DNA methylation in human cancer cells induces BORIS expression and an altered histone H3 modification pattern at the BORIS promoter [51]. Consistently, multiple BORIS isoforms are activated synergistically by DAC + trichostatin A (TSA) treatment, indicating that both DNA hypomethylation and histone acetylation are sufficient for BORIS isoform expression in EOC cells [58]. Regardless, studies on the effect of histone modification on CT gene expression are limited, and most studies thus far have typically applied a pharmacological approach. Evidence based on direct detection of specific histone modifications in ovarian cancer tissues is not currently available. Further studies are needed to provide clarity on this topic.

**Effect of copy number alteration (CNA) on activation of CT genes**

Copy number aberrations are identified as remarkable molecular alterations in EOC and have the ability to alter gene expression [5]. Based on cBioPortal analysis of TCGA data [59], one recent study demonstrated that CNA was correlated with PRAME expression in high-grade serous ovarian cancer (HGSOC). Notably, the proportion of PRAME amplification was low compared with the high prevalence of increased PRAME expression [60]. Another study reported that among TCGA HGSOC data, CT45 expression did not correlate with copy number status [31]. Therefore, CNA may have a minor contribution to the observed increased expression of CT genes in ovarian cancer.

## Effect of TP53 mutation on CT gene expression

TP53 mutations are present in almost all HGSOCs [5, 7], a finding that has prompted investigation into whether a relationship exists between CT gene expression and TP53 mutation status in serous ovarian cancer. Devor *et al* presented evidence that PLAC1 transcriptional repression occurs in serous ovarian carcinomas only in the presence of wild-type TP53, whereas mutant or absent TP53 protein de-represses PLAC1 transcription [61]. These authors proposed that the inability of mutant TP53 to occupy the TP53 binding site in the PLAC1 P1 promoter allowed transcription to occur [61]. By directly binding to the promoter or interacting with the transcription factor Sp1, expression of wild-type TP53 protein represses the activity of all BORIS promoters [62]. Therefore, as a gatekeeper of genome instability, TP53 mutations may contribute to activation of CT genes. Another comprehensive study demonstrated CT genes to be mutually exclusively correlated with somatic mutations in significantly mutated genes (SMG) in cancers, indicating that these CT genes may function as novel drivers that complement known mut-driver genes [9].

## CT gene function in ovarian cancer

Based on a multidimensional functional genomics approach, Maxfield *et al* uncovered that multiple CT genes play an obligatory role in multiple hallmarks of cancer and function as pathological drivers of major pathways, including HIF, WNT and TGFb signaling [63]. Cellular switching from a specific somatic designation to acquisition of a germline cell-like state (soma-to-germline transition) is the key feature of oncogenesis, and CT genes play a functional role in initiating and maintaining oncogenesis [64].

Although the function of many CT genes in ovarian cancer remains unclear, it appears that these genes may exert oncogenic functions in tumorigenesis and progression. Heat shock protein 70-2 (HSP70-2) is required for the formation of an active CDC2/Cyclin B complex in meiosis I division during spermatogenesis [65]. Moreover, HSP70-2 knock-out mice fail to complete meiosis [65]. Several reports have demonstrated that HSP70-2 is involved in cellular proliferation and the early spread of various malignancies, including breast cancer [66] and ovarian cancer [67]. Using *in vitro* and *in vivo* human ovarian xenograft mouse models, one recent study demonstrated that ablation of HSP70-2 resulted in cell cycle arrest, onset of senescence and apoptosis and inhibited cell proliferation, cell viability and colony formation in EOC [67]. In addition, HSP70-2-depleted EOC cells exhibit downregulation of anti-apoptotic proteins and increased expression of pro-apoptotic genes, subsequently leading to upregulation of cytochrome-C, caspase 3, caspase 7 and caspase 9 [67]. Therefore, HSP70-2 may promote malignancy in EOC cells.

AKAPs comprise a family of scaffolding proteins that anchor different proteins and tether protein kinase A (PKA) at specific locations to restrict enzymatic activities [68]. Some of the AKAPs considered to be CT genes in ovarian cancer include AKAP3 [42] and AKAP4 [29]. These proteins typically bind to the regulatory subunit RI or RII domain of PKA and cooperatively terminate cAMP signaling [68]. Notably, PKA activity and anchoring are required for ovarian cancer cell migration and invasion [69]. Kumar *et al* demonstrated that AKAP4 knockdown inhibits cell proliferation and viability, induces cell cycle arrest, and increases reactive oxygen species (ROS) generation, DNA damage and apoptosis in ovarian cancer cells and reduces tumor growth in an ovarian cancer xenograft model [70]. Furthermore, AKAP4 knockdown inhibits wound healing and reduces metastatic markers in ovarian cancer cells [70].

The primary function of Sp17, a protein originally reported to be expressed exclusively in the testis, is binding to the zona pellucida [71]. One study reported that the metastatic ascitic fluid from eight EOC patients was positive for Sp17 [32], and two others consistently found that migration of ovarian cancer cells (HO-8910) was substantially enhanced after Sp17 overexpression [32, 72]. Several reports have demonstrated that Sp17 plays an important role in cell-cell adhesion and/or cell migration in transformed, lymphocytic, and hematopoietic cells, possibly through its interaction with extracellular heparin sulfate or binding to AKAP3 [73, 74]*.* Another study demonstrated that an Sp17-based vaccine induced an enduring defense response against ovarian cancer development in C57BL/6 mice with ID8 cells following prophylactic and therapeutic treatments [75].

HORMAD1, a meiosis I checkpoint protein that is expressed during gonadal development, is upstream of ATM kinase (a serine-threonine protein kinase) activation [76]. In ovarian cancer cells, HORMAD1 siRNA enhanced docetaxel-induced apoptosis and reduced invasive and migratory capacities. In addition, HORMAD1 targeting *in vivo* with siRNA-DOPC significantly reduced tumor weight and ascites production, in association with substantial reductions in angiogenesis and VEGF and NF-κB levels [77]. Collectively, re-expression of CT genes results in crucial biological activities in ovarian cancer.

## Chemotherapy resistance

Chemotherapy is widely applied for the treatment of ovarian cancer, but drug resistance remains a major obstacle for success. Indeed, some evidence suggests that drug resistance is associated with increased expression of a variety of CT genes. One study demonstrated that MAGE gene families are overexpressed in some drug-resistant ovarian cell lines and that transfection of MAGE-A2 and MAGE-A6 into a sensitive cell line can facilitate cell growth and induce paclitaxel and doxorubicin resistance [78]. Another study indicated that overexpression of MAGE-A3 correlates with a doxorubicin-resistant phenotype [79]. In addition, MAGE-C1 expression was associated with platinum-sensitive disease and clinical response [21]. For other CT genes, high PLU-1 levels are significantly associated with chemotherapy resistance, and PLU-1 is an independent factor for chemotherapy resistance in patients with EOC [80]. TRAG-3, which was originally identified as a Taxol resistance-associated gene from an ovarian carcinoma cell line, is associated with the chemotherapy-resistant and neoplastic phenotype [81]. Notably, Sp17 overexpression was found to reduce the chemosensitivity of ovarian carcinoma cells to carboplatin and cisplatin [32, 36]. Importantly, PIWIL2 is required for maintaining the equilibrium between euchromatin and heterochromatin in response to cisplatin treatment in mammalian cells, and increased levels of PIWIL2 expression in human ovarian cancer cell lines (CDDP and 2008C13) facilitate cisplatin resistance, likely through enhanced repair of cisplatin-induced DNA damage [82]. In a pan-genomic siRNA screen for modifiers of chemo-responsiveness, Whitehurst *et al.* identified that OY-TES-1, a specifier of paclitaxel resistance, is necessary and sufficient for paclitaxel resistance in ovarian cancer cell lines and ovarian tumor explants [83]. Although the researchers hypothesized that overexpression of these genes contributes to the chemo-resistant phenotype via co-expression with multidrug resistance genes [50, 78, 80], the detailed mechanism by which CT genes induce drug resistance requires further investigation.

## Application in immunotherapy

In addition to traditional operation or chemotherapy, a subset of burgeoning cure modalities, including target therapy and radiation therapy, aims to abrogate tumor cells, and CT genes may represent a class of potential targets for tumor immunotherapy due to their specific expression patterns and oncogenic function. These genes also respond to T-cells and subsequently stimulate the immune response. Some NY-ESO-1 clinical trials thus far have been carried out in ovarian cancer patients, and the indicated NY-ESO-1 targeted therapy has a potential clinical benefit [13]. For example, the antigen was able to induce cellular and humoral immune responses in a high proportion of patients with NY-ESO-1 expression. Odunsi *et al* reported that NY-ESO-1_157–170_ with dual HLA class I and II specificities should elicit integrated Ab, HLA-DP4-restricted CD4+ T cell and HLA-A2 and A24-restricted CD8+ T cell responses in ovarian cancer patients [84]. Seven years later, these researchers described an encouraging result that a combination of decitabine and the NY-ESO-1 vaccine resulted in disease stabilization or a partial clinical response in relapsed EOC patients [85] Decitabine may enhance the response to the NY-ESO-1 vaccine by promoting CTA promoter hypomethylation and immunity [86–88]. This regimen complements standard second-line chemotherapy. Another CT antigen vaccine undergoing assessment in clinical trials is MAGE-A3, which is frequently expressed in various cancers [89]. However, there are currently no ongoing clinical studies of MAGE-A3-directed anti-ovarian cancer immunotherapies. In addition, Chiriva-Internati *et al* demonstrated that HLA class I-restricted Sp17-specific cytotoxic T-lymphocytes (CTLs) enable autologous tumor killing through the perforin pathway [90, 91]. To date, two studies have confirmed the different formulations of the Sp17 vaccine in a murine ovarian cancer model. One vaccine was CpG-adjuvated Sp17, which inhibited tumor growth and prolonged the OS of vaccinated mice [92]. The other vaccine was the nanoparticle-based hSp_17111–142_ peptide, which induced mixed Th1/Th2 responses and subsequently stimulated B-cell production of IgG1 and IgG2a. This vaccine exhibited advantages leading to the promotion of immunogenicity and strong cross-reactivity of the antibody [93]. Nonetheless, clinical trials on ovarian cancer patients must be initiated to verify whether the Sp17 vaccine exhibits treatment efficacy in humans.

## Conclusions

In summary, our current understanding of the expression, mechanism and function of CT genes in ovarian cancer is still largely in the early stages. Given advances in whole-genome sequencing and shared large databases, such as Gene Expression Omnibus (GEO) and TCGA, further studies on the characteristic features of CT genes in testis and ovarian cancers are warranted to provide insight into similarities and differences. More importantly, the unique properties of CT genes, such as their inherent immunogenicity and heterogeneity in their expression patterns, indicate that they have great potential for clinical application. To better understand the immunological processes of CT genes in ovarian cancer, more clinical trials directed against CT antigens and the development of combination therapies are needed. Such efforts will contribute to targeted immunotherapies for ovarian cancer.

## Abbreviations

CT: Cancer-testis
CTAs: Cancer-testis antigens
DAC: 5-aza-2'-deoxycytidine
EOC: Epithelial ovarian cancer
FIGO: International Federation of Gynecology and Obstetrics
GEO: Gene Expression Omnibus.
GTEx: The Genotype-Tissue Expression
HDAC: Histone deacetylase
HGSOC: High-grade serous ovarian cancer
HSP70-2: Heat shock protein 70-2
IHC: Immunohistochemistry
NER: Nucleotide excision repair
OS: Overall survival
PFS: Progression-free survival
PKA: Protein kinase A
RT-PCR: reverse transcription-PCR
SMG: Significantly mutated genes
Sp17: Sperm protein antigen 17
TCGA: The Cancer Genome Atlas
